# An Efficient Electrochemical Sensor Driven by Hierarchical Hetero-Nanostructures Consisting of RuO_2_ Nanorods on WO_3_ Nanofibers for Detecting Biologically Relevant Molecules

**DOI:** 10.3390/s19153295

**Published:** 2019-07-26

**Authors:** Hyerim Lee, Yeomin Kim, Areum Yu, Dasol Jin, Ara Jo, Youngmi Lee, Myung Hwa Kim, Chongmok Lee

**Affiliations:** Department of Chemistry & Nanoscience, Ewha Womans University, Seoul 03760, Korea

**Keywords:** ruthenium dioxide, tungsten trioxide, nanofibers, nanorods, electrochemical sensors

## Abstract

By means of electrospinning with the thermal annealing process, we investigate a highly efficient sensing platform driven by a hierarchical hetero-nanostructure for the sensitive detection of biologically relevant molecules, consisting of single crystalline ruthenium dioxide nanorods (RuO_2_ NRs) directly grown on the surface of electrospun tungsten trioxide nanofibers (WO_3_ NFs). Electrochemical measurements reveal the enhanced electron transfer kinetics at the prepared RuO_2_ NRs-WO_3_ NFs hetero-nanostructures due to the incorporation of conductive RuO_2_ NRs nanostructures with a high surface area, resulting in improved relevant electrochemical sensing performances for detecting H_2_O_2_ and L-ascorbic acid with high sensitivity.

## 1. Introduction

Recently, a variety of metal oxide nanostructures have been extensively utilized as efficient electrode substances owing to their outstanding electrocatalytic properties. Among them, ruthenium dioxide (RuO_2_) has been well described as one of the best electrocatalysts for diverse energy related applications, such as the hydrogen evolution reaction (HER), oxygen evolution reaction (OER), and supercapacitors because of its high electric conductivity, catalytic activity, and thermal stability [1,2,3]. Especially, RuO_2_ has been used as an efficient electrode system for supercapacitors owing to its excellent charging-discharging behavior [1,4,5,6,7,8]. Generally, RuO_2_ as a promising catalytic material is often used in the forms of hybrid structures or alloys with other abundant transition metals in consideration of the relatively high cost of RuO_2_. Thus, there have been previous reports regarding the use of RuO_2_ nanostructures with other metal oxides as supercapacitors [9,10,11,12], and biosensing applications [1,2,3,13,14].

Tungsten trioxide (WO_3_) nanostructures have been also extensively studied in various applications due to its earth-abundance, high durability, and chemical stabilities in aqueous acid media, as well as good electrochemical conductivity [15,16,17,18]. Thereby it has been developed as a catalyst for the hydrogen evolution reaction (HER) and supercapacitors in an acidic solution [19,20,21,22]. WO_3_ also constitutes composites with other novel metals like Pt [23,24,25,26], Ir [17,23,27], and Ru [16,28,29,30], or supporting materials.

Nanostructured catalysts are applied to nonenzymatic electrochemical biosensors. Electrochemical properties can be enhanced from the increase of active surfaces. The detection of hydrogen peroxide (H_2_O_2_) is important in not only biomedical and environmental applications, but also in the enzymatic system [31]. While ascorbic acid (AA) has an important role in the physiological function of organisms, a deficiency of AA causes several diseases [32,33]. Therefore, the detection and accurate quantification of target material with selectivity is highly required.

In this study, we introduce a facile fabrication of hybrid nanostructures consisting of single crystalline RuO_2_ nanorods on eletrospun WO_3_ nanofibers by utilizing electrospinning and thermal annealing processes. In addition, the fundamental electrochemical performances of RuO_2_ nanorods-WO_3_ nanofibers (RuO_2_ NRs-WO_3_ NFs) are carefully investigated, which confirm their characteristics of fast electron-transfer reactions and possibility as a catalytic sensing platform for detecting l-ascorbic acid (AA) and hydrogen peroxide (H_2_O_2_) in phosphate buffered solution (PBS).

## 2. Materials and Methods

Tungsten chloride (WCl_6_, ≥ 99.9% true metal basis), ruthenium chloride hydrate (RuCl_3_∙*x*H_2_O, 99.98% trace metal basis), poly(vinyl pyrrolidone) (PVP, MW = 1,300,000), N,N-dimethylformamide (DMF), potassium ferricyanide (K_3_[Fe(CN)_6_]), l-ascorbic acid (AA), 4-acetamidophenol (AP), dopamine hydrochloride (DA), uric acid (UA), d-(+)-glucose, hydrogen peroxide (H_2_O_2_, 35 wt% solution in water), sodium phosphate monobasic, and sodium phosphate dibasic were supplied by Sigma Aldrich (St. Louis, MO). Commercial Pt/C and Ir/C (both of them were 20 wt% each metal loading on Vulcan XC-72) were obtained from E-TEK Company. Sulfuric acid (H_2_SO_4_) and acetic acid were provided by Ducksan (Korea). Sodium hydroxide (NaOH) was purchased from Daejung (Korea). Deionized water with resistivity ≥ 18 MΩ∙cm was used in all processes.

First, WO_3_ nanofibers were synthesized by electrospinning and thermal annealing process according to the reported method [23]. To prepare electrospinning solution, 1.5 g WCl_6_ were dissolved in 10.549 mL DMF with 1.25 g PVP and 0.191 mL acetic acid. After being stirred overnight, the solution was loaded into syringe and applied to the needle of the electrospinning system (Nano NC ESR 200R2). The needle was connected to a voltage power supply (applied voltage = 17.5 kV) at a flow rate of 5 μL/min, and the distance from needle tip to aluminum plate to collect as spun NFs was 15 cm. The collected electrospun NFs were calcinated at 500 °C for 1 h under a mixed gas atmosphere of 80 sccm of He and 10 sccm of O_2_ with ramping rate of 1 °C/min.

Ruthenium hydroxide (Ru(OH)_3_) precursor was prepared by a precipitation process via the acid-base reaction with controlling pH of aqueous solution. The pH of the final precursor solution at about pH 10 was carefully achieved by slowly dropping 0.1 M NaOH dilute solution into 5 mM RuCl_3_∙*x*H_2_O aqueous solution [2,13]. After precipitation, the precursor solution was washed five times with deionized water, and then re-dispersed in 2~3 mL pure deionized water again. To grow RuO_2_ NRs on WO_3_ NFs, 2 mg of WO_3_ NFs was dispersed into 1 mL deionized water and then mixed with 1 mL Ru(OH)_3_ precursor solution. After sonication for 30 min, the mixed solution was directly dropped on the center of Si wafer. WO_3_ nanofibers containing Ru(OH)_3_ precursors loaded on the Si wafer was placed into the center of a furnace and calcined at 300 ℃ for 5 h in air. The furnace was then allowed to cool to room temperature.

The surface morphology of as-grown products was examined by field emission scanning electron microscopy (FE-SEM; JEOL JSM-6700F). The detailed crystal structures were also investigated by a high-resolution transmission electron microscopy (HRTEM, Cs-corrected STEM, JEOL JEM-2100F) instrument equipped with selected area electron diffraction (SAED) micrographs and elemental EDX mapping with a Tecnai-F20 system operated at 200 kV. Additionally, high resolution X-ray diffraction measurement (XRD; Bruker D8 DISCOVER, Cu Kα radiation), and X-ray photoelectron spectroscopy (XPS; Theta Probe AR-XPS System. Al Kα radiation) were performed to investigate the crystal structure and surface binding energies of as-grown RuO_2_ NR-WO_3_ NFs.

For electrochemical measurements, a three-electrode system was used with a modified glassy carbon (GC) electrode (3 mm in diameter), a saturated calomel electrode (S.C.E.), and a coiled Pt wire (1 mm in diameter, length immersed in a solution ~ 10 cm) as the working electrode, the reference electrode, and the counter electrode, respectively. All electrochemical experiments conducted with CHI 650E workstation (CH Instruments) and BAS100B (BAS Inc.). To modify the surface of a GC electrode with synthesized nanomaterials, 2 mg of RuO_2_ NR-WO_3_ NFs was suspended in 1.0 mL deionized water. Subsequently, 10 μL of the solution were dropped onto the GC electrode surface three times. Then, 10 μL of 0.05 wt% Nafion solution were loaded onto the modified GC electrode surface. Cyclic voltammetry (CV) measurements was used for analyze the capacitive behavior in 1 M H_2_SO_4_. For sensing experiments, linear sweep voltammetry (LSV) was also used with rotating disk electrode (RDE) at a scan rate of 5 mV s^−1^ with rotating speed of 1600 rpm, and amperometry measurements were used in 0.1 M phosphate buffered saline (PBS) at physiological condition pH (7.4).

## 3. Results and Discussion

### 3.1. Synthesis of Hybrid Nanostructures of RuO_2_ Nanorods on Electrospun WO_3_ Nanofibers

**Figure 1:**
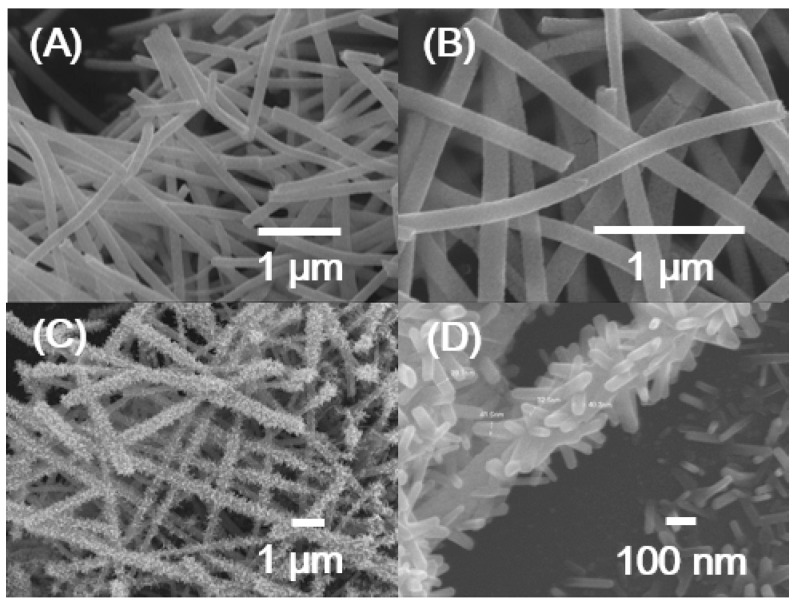
(**A**,**B**) SEM images of pure electrospun WO_3_ nanofibers (WO_3_ NFs). (**C**,**D**) SEM images of as grown RuO_2_ nanorods on the electrospun WO_3_ nanofibers (RuO_2_ NRs-WO_3_ NFs).

Figure 1A,B show FE-SEM images of WO_3_ NFs annealed at 500 °C. The calcined WO_3_ NFs revealed a very fine structure and the diameter of the fibers was around 200 nm. On the other hand, after the heat treatment of the mixed solution composed of Ru(OH)_3_ precursors and WO_3_ NFs at 300 ℃ for 5 h, it is readily identified that RuO_2_ NRs were directly grown on the electrospun WO_3_ NFs as shown in Figure 1C,D. Figure 1D represents the as grown RuO_2_ NRs covering the entire surface of WO_3_ NFs. The lateral dimension of RuO_2_ NRs is estimated to be about 40 nm and the length up to 300 nm. Careful EDS measurements indicate that the atomic ratio of Ru to W is confirmed as 45:55. According to our previous real-time study by in situ synchrotron XRD, a simple recrystallization process by thermal annealing might be responsible for the growth mechanism of RuO_2_ NRs. It was carefully suggested that Ru diffusion to the amorphous nanoparticles followed by diffusion to the growing surface of the nanorod plays an essential role in the growth of RuO_2_ NRs in oxygen ambient, which is supported by the nucleation theory [34].

**Figure 2:**
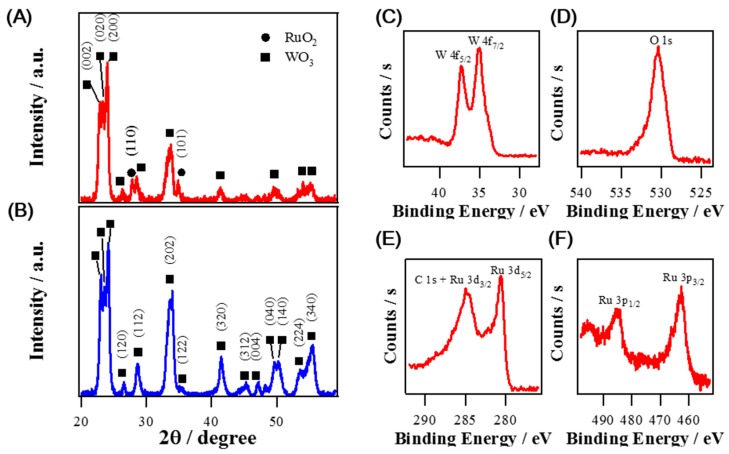
(**A**) XRD spectrum for RuO_2_ NRs-WO_3_ NFs (**B**) XRD spectrum for pure WO_3_ NFs. (**C**–**F**) high resolution XPS spectra for RuO_2_ NRs-WO_3_ NFs, (**C**) W 4f, (**D**) O 1s, (**E**) Ru 3d, and (**F**) Ru 3p regions, respectively.

Figure 2 represents XRD spectra and high resolution XPS spectra of composite RuO_2_ NRs-WO_3_ NFs and pure WO_3_ NFs. XRD spectrum of pure WO_3_ NFs in Figure 2B demonstrates that all peaks are closely matched with the monoclinic phase of WO_3_ [19,35]. On the other hand, XRD spectrum of composite RuO_2_ NR-WO_3_ NFs confirms the same monoclinic phase WO_3_ peaks including two major peaks at 27.1° and 34.8° corresponding to (110) and (101) crystallographic planes of tetragonal RuO_2_ structure as displayed in Figure 2A [2,13]. To investigate the oxidation states of Ru, W, and O atoms, XPS measurements were performed. In Figure 2C, two separated binding energies at 35.1 eV and 37.3 eV are clearly identified as two spin-orbit states of W 4f_5/2_ and W 4f_7/2_, respectively, which indicates the oxidation state of +6 for W in WO_3_ NFs [16,36]. Both high resolution Ru 3d and Ru 3p spectra were shown in Figure 2E,F. Although the peak position of Ru 3d_3/2_ is overlapped with C 1s [16,37], the oxidation state of Ru species is readily identified to Ru^4+^ based on the binding energies of 280.7 eV and 462.8 eV, indexed to Ru 3d_5/2_ and Ru 3p_3/2_, respectively [37,38]. In addition, the peak at 530.5 eV of O 1s is associated with O^2−^ in RuO_2_ and WO_3_ metal oxides as shown in Figure 2D.

**Figure 3:**
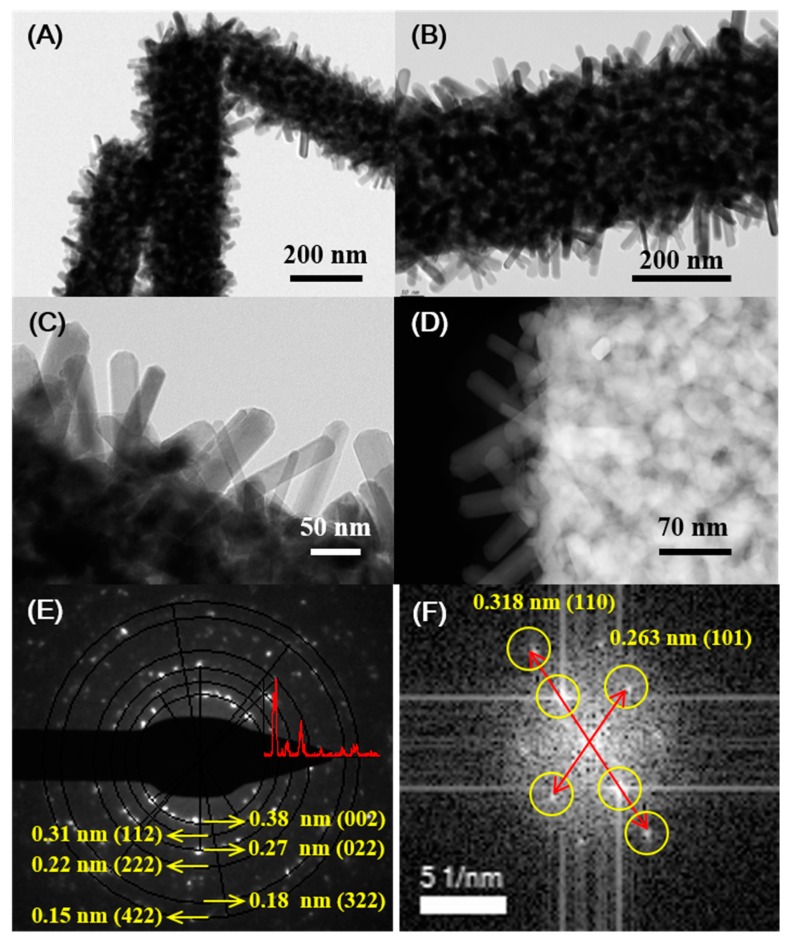
(**A**,**B**) low magnification TEM images for RuO_2_ nanorods on a single WO_3_ nanofiber. (**C**) The high resolution TEM image for RuO_2_ nanorods on a single WO_3_ nanofiber. (**D**) The bright field TEM image for RuO_2_ nanorods on a single WO_3_ nanofiber. (**E**,**F**) SAED pattern for a WO_3_ nanofiber and fast Fourier transform (FFT) of the lattice-resolved image for a single RuO_2_ nanorod.

Figure 3 indicates TEM images and SAED pattern for a single WO_3_ nanofiber covered with RuO_2_ nanorods. As shown in Figure 3A,B, low-magnification TEM images show the high density of RuO_2_ nanorods directly grown on the porous surface of WO_3_ nanofiber. The SAED pattern shown in Figure 3E reveals the existence of many different crystalline phases in a WO_3_ nanofiber which confirms the polycrystalline nature of a WO_3_ nanofiber. On the contrary, the fast Fourier transform (FFT) of the lattice-resolved image for a RuO_2_ nanorod in Figure 3F represents highly ordered lattice fringes with a single crystal nature. The values of lattice spacing of adjacent planes are estimated by about 0.318 nm and 0.263 nm, corresponding to those of between the (110) planes and (101) for the tetragonal RuO_2_, respectively. Furthermore, TEM-EDS element mapping analysis from the high-angle annular dark field (HAADF) STEM image shown in Figure S1 confirms the homogenous distribution of Ru, W, and O in distinct regions in the hierarchical nanostructure. W atoms exist on the backbone of the nanofibers, whereas Ru atoms exclusively exist on the branched nanorods. Oxygen atoms exist both on the backbone of the nanofibers and branched nanorods. Thus, we successfully fabricate the high density of single-crystalline RuO_2_ nanorods on WO_3_ nanofibers by using a combination of an electrospinning process and a thermal annealing process. Our growth process thus provides a simple methodology for the fabrication of highly efficient electrocatalysts.

### 3.2. Electrochemical Properties for Capacitive Behaviors of RuO_2_ NRs-WO_3_ NFs

The general electrochemical activities of RuO_2_ NRs-WO_3_ NFs and WO_3_ NFs were examined by CV in 10 mM [Fe(CN)_6_]^3−^ aqueous solution containing 1 M KCl. Figure S2 displays CV curves of RuO_2_ NRs-WO_3_ NFs and WO_3_ NFs at a scan rate 100 mV s^−1^. Voltammetric current peaks at RuO_2_ NRs-WO_3_ NFs are reversible, while those of WO_3_ NFs are quasi-reversible. It seems to be ascribed to that RuO_2_ NRs-WO_3_ NFs allow very facile heterogeneous electron transfer kinetics with high electric conductivities in contrast to WO_3_ NFs. Moreover, RuO_2_ NRs-WO_3_ NFs show a much larger charging current in CV than WO_3_ NFs.

**Figure 4:**
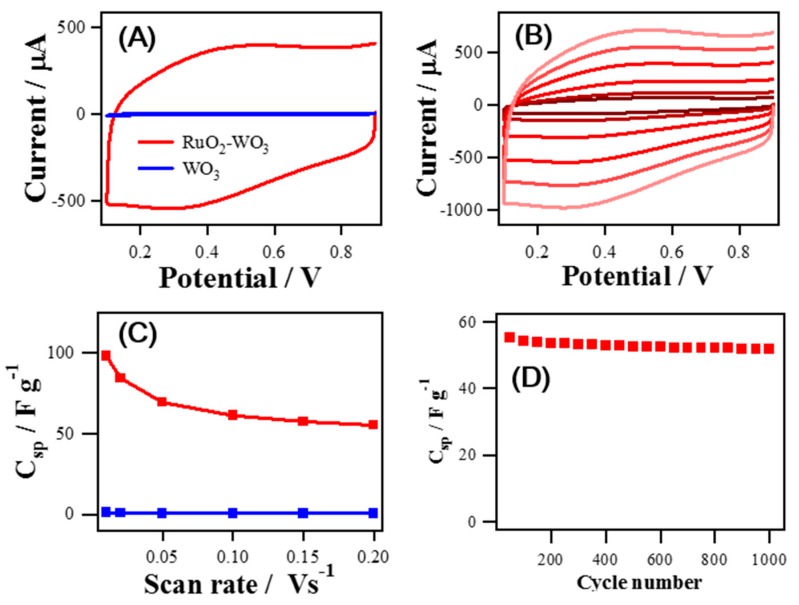
Capacitive current measurements in 1 M H_2_SO_4_ solution for (**A**) RuO_2_ NRs-WO_3_ NFs and WO_3_ NFs at a scan rate 100 mV s^−1^, and (**B**) RuO_2_ NRs-WO_3_ NFs with varing the scan rate from 10 mV s^−1^ to 200 mV s^−1^. (**C**) Changes of specific capacitance (C_sp_) values of RuO_2_ NRs-WO_3_ NFs and WO_3_ NFs as a function of the CV scan rate (from 10 mV s^−1^ to 200 mV s^−1^). (**D**) Plot of the C_sp_ values of RuO_2_ NRs-WO_3_ NFs depending on the number of repeated CV cycles in 1 M H_2_SO_4_.

To characterize the charging behavior of the synthesized materials, CV was measured for a potential range from 0.1 V to 0.9 V (vs. S.C.E.) in 1 M H_2_SO_4_ as seen in Figure 4. Figure 4A shows CV results comparing RuO_2_ NRs-WO_3_ NFs and WO_3_ NFs at a scan rate 100 mV s^−1^. It supports the enhanced capacity of RuO_2_ NRs-WO_3_ NFs as the RuO_2_ NRs were grown on WO_3_ NFs. To examine the charging performance, the average specific capacitance values (C_sp_, F g^−1^) were calculated with the following Equation (1) using CV curves shown in Figure 4B.

(1) $$C_{\mathrm{sp}}=\frac{1}{2\times v\times ∆m\times ∆V}\;\int IdV$$

where *v* is the scan rate (V s^−1^), ∆m is the weight of electrode materials, ∆V is the potential range, and IdV is the area under CV curve [39]. At the scan rate of 10 mV s^−1^, the C_sp_ values of the synthesized materials, RuO_2_ NRs-WO_3_ NFs and WO_3_ NFs, are 98.15 F g^−1^ and 0.95 F g^−1^, respectively. The C_sp_ of RuO_2_ NRs-WO_3_ NFs is obviously 103-fold higher than that of WO_3_ NFs as shown in Figure 4C. As the scan rate increases, C_sp_ becomes smaller and the C_sp_ of RuO_2_ NRs-WO_3_ NFs and WO_3_ NFs decreased down to 57% and 42%, respectively, while increasing the scan rate from 10 mV s^−1^ to 200 mV s^−1^. This additionally indicates the successful decoration of WO_3_ NFs with RuO_2_ NRs forming the hierarchical hetero-nanostructures.

Electrochemical impedance spectroscopy (EIS) was also employed to examine the electrochemical behavior of RuO_2_ NRs-WO_3_ NFs and WO_3_ NFs. EIS measurement was carried out at 0.5 V (vs. S.C.E.) under the same condition of CV experiments with a frequency range of 0.1 Hz–1000 kHz as shown in Figure S3. The Nyquist plot of RuO_2_ NRs-WO_3_ NFs was closer to a vertical line than that of WO_3_ NFs, exhibiting nearly pure capacitive behavior of RuO_2_ NR-WO_3_ NFs [1,40]. The stability of RuO_2_ NRs-WO_3_ NFs for capacitance was demonstrated by monitoring the change of C_sp_ during repeated CV cycles as depicted in Figure 4D. RuO_2_ NRs-WO_3_ NFs excellently maintained about 96% of its original C_sp_ for the 1000 CV cycles at a scan rate of 100 mV s^−1^.

### 3.3. Applications to Electrochemical Sensing of AA and H_2_O_2_

**Figure 5:**
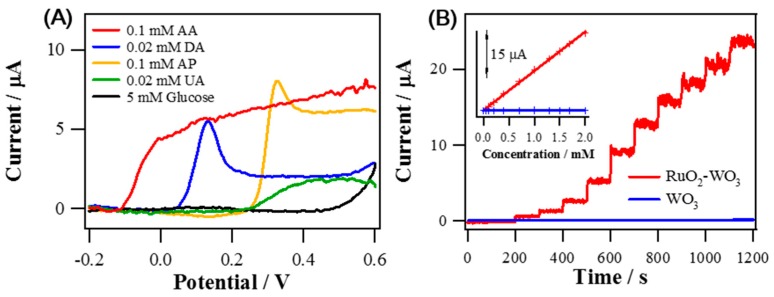
(**A**) Background-corrected LSVs of RuO_2_ NRs-WO_3_ NFs obtained in 0.1 M PBS (pH 7.4) independently containing one of 0.1 mM AA, 0.02 mM DA, 0.1 mM AP, 0.02 mM UA, 5 mM glucose (scan rate of 5 mV s^−1^; and rotating speed of 1600 rpm). (**B**) Amperometric current responses of RuO_2_ NRs-WO_3_ NFs and WO_3_ NFs for successive AA standard solution injections to increase the AA bulk concentration from 5 μM to 2 mM in 0.1 M PBS (pH 7.4) with *E*_app_ = 0 V (vs. S.C.E.). The inset: The calibration curves showing the current responses vs. concentration.

The electrochemical properties of RuO_2_ NRs-WO_3_ NFs for AA oxidation were also studied. LSV measurements in 0.1 M PBS were used for examining the oxidations of various biomaterials such as AA, DA, UA, AP, and glucose. The chosen concentrations are slightly above the physiological concentrations. As shown in Figure 5A, AA oxidation started to occur from the most negative potential compared with other biomaterials. Amperometric measurements of RuO_2_ NRs-WO_3_ NFs and WO_3_ NFs were conducted at 0 V (vs. S.C.E.) which possibly allow for the oxidation of AA only, excepting for the other tested biomolecules as seen in the LSV results of Figure 5A.

**Figure 6:**
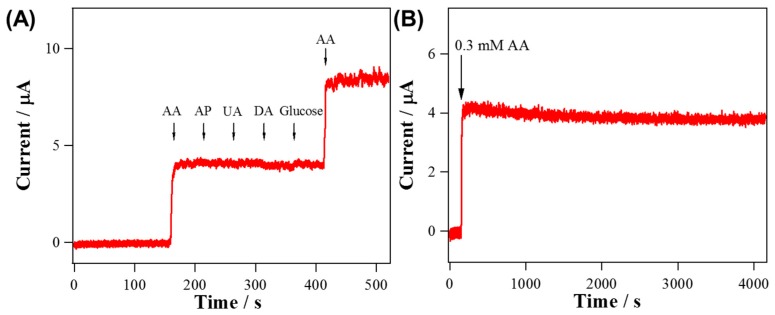
(**A**) Amperometric response of RuO_2_ NRs-WO_3_ NFs to sequential additions of 0.3 mM AA, 0.1 mM AP, 0.1 mM UA, 0.1 μM DA, 5 mM glucose and 0.6 mM AA to 0.1 M PBS (pH 7.4) with *E*_app_ = 0 V (vs. S.C.E.). (**B**) Continuous amperometric response of RuO_2_ NRs-WO_3_ NFs to 0.3 mM AA in 0.1 M PBS during 4200 s with *E*_app_ = 0 V (vs. S.C.E.).

As observed in Figure 5B, the anodic currents of both electrodes were increased linearly with the concentration of AA increased from 5 μM to 2 mM. Also, the calibration curves based on the amperometric data were depicted in inset of Figure 5B. The sensitivity of RuO_2_ NRs-WO_3_ NFs (171.7 μA mM^−1^ cm^−2^, R^2^ = 0.9990, normalized to GC substrate electrode area, 0.072 cm^2^) were surprisingly increased by 244 times compared to that of WO_3_ NFs (0.704 μA mM^−1^ cm^−2^, R^2^ = 0.9990). Most of the typical biological samples are complex, having various oxidizable species, so selectivity to a targeted analyte is an essential requirement for any sensor. In Figure 6A, current responses for AA oxidation were stable against the additions of 0.1 mM AP, 0.1 mM UA, 0.1 μM DA and 5 mM glucose at 0 V. Additionally, the stability of RuO_2_ NRs-WO_3_ NFs was measured by monitoring the change of current at 0 V in 0.1 M PBS containing 0.3 mM AA. The amperometric response of RuO_2_ NRs-WO_3_ NFs retained 96% of the initial current level during over a 4200-s measurement in Figure 6B, supporting its excellent stability. Table 1 summarizes the properties of RuO_2_ NRs-WO_3_ NFs in comparison with other Ru-based materials used as AA sensors.

**Figure 7:**
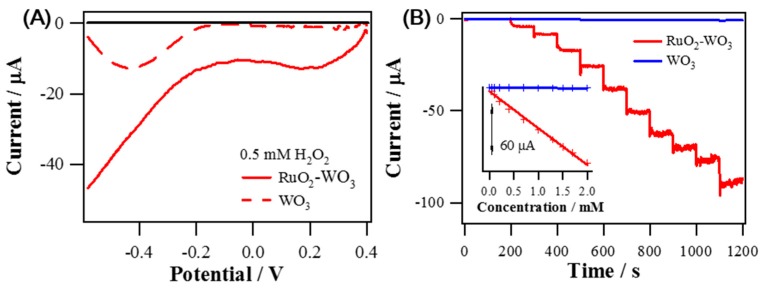
(**A**) Background-corrected LSVs of RuO_2_ NRs-WO_3_ NFs and WO_3_ NFs obtained in 0.1 M PBS (pH 7.4) containing 0.5 mM H_2_O_2_ with a scan rate of 5 mV s^−1^, at an electrode rotating speed of 1600 rpm. (**B**) Amperometric current responses of RuO_2_ NRs-WO_3_ NFs and WO_3_ NFs to successive H_2_O_2_ injections from 0.005 mM to 2 mM in 0.1 M PBS (pH 7.4) at −0.2 V (vs. S.C.E.); the inset: corresponding calibration curves.

The catalytic effect of RuO_2_ NRs-WO_3_ NFs for H_2_O_2_ reduction was also measured. Figure 7A shows overlaid LSV results of RuO_2_ NRs-WO_3_ NFs and WO_3_ NFs. It presents clearly that H_2_O_2_ reduction at RuO_2_ NRs-WO_3_ NFs starts from a much less negative potential with much greater reduction current level than that at WO_3_ NFs. In fact, the cathodic current level measured at −0.2 V (vs. S.C.E.) was more greatly increased for RuO_2_ NRs-WO_3_ NFs than WO_3_ NFs in response to the successive increase of H_2_O_2_ concentration (Figure 7B). Inset of Figure 7B shows the calibrated current vs concentration with good linearity. Obtained sensitivities from the calibration curves are 619.7 μA mM^−1^ cm^−2^ (R^2^ = 0.9960), and 5.5 μA mM^−1^ cm^−2^ (R^2^ = 0.9384) for RuO_2_ NRs-WO_3_ NFs and WO_3_ NFs, respectively. Sensitivity of RuO_2_ NR-WO_3_ NFs is 112-fold higher than the value of WO_3_ NFs, and therefore it supports the enhanced activities of RuO_2_ NRs-WO_3_ NFs toward H_2_O_2_ reduction. The H_2_O_2_ reduction current instead of the oxidation current was monitored to sense H_2_O_2_ in order to avoid the interference from many oxidizable species generally present in biological systems. Figure S4 represents the selectivity of RuO_2_ NRs-WO_3_ NFs for H_2_O_2_ reduction. The current responses of RuO_2_ NRs-WO_3_ NFs at −0.2 V (vs. S.C.E.) for H_2_O_2_ reduction were obvious; however, there were no noticeable responses to the successive injections of other biological materials: 0.1 mM AA, 0.1 mM UA, 0.1 μM DA, 5 mM glucose, 0.1 mM AP, and 30 μM O_2_. RuO_2_ NR-WO_3_ NFs show relatively excellent catalytic activities for H_2_O_2_ reduction compared to other previous Ru-based materials as compared in Table 2. RuO_2_ NRs-WO_3_ NFs for measuring H_2_O_2_ reduction current was less stable than that for AA oxidation. In fact, H_2_O_2_ reduction current measured at −0.2 V was decreased to ~60% of the initial current level after 4200-s continuous measurement (data not shown).

## 4. Conclusions

We report the successful fabrication of single crystalline RuO_2_ nanorods on WO_3_ nanofibers by electrospinning and calcination. Microscopic and spectroscopic measurements such as SEM with EDS, XRD, and XPS were used to characterize the structure and composition of RuO_2_ NRs-WO_3_ NFs. The RuO_2_ NRs-WO_3_ NFs showed improved electrocatalytic activities over WO_3_ NFs through a series of electrochemical measurements. In 1 M H_2_SO_4_ solution, RuO_2_ NRs-WO_3_ NFs represent a higher C_sp_, 98.15 F g^−1^, by 103-fold with good stability and a sharper slope than pure WO_3_ NFs. Additionally, the RuO_2_ NRs-WO_3_ NFs have dramatically enhanced sensing abilities, in accordance with 224 times (171.7 μA mM^−1^ cm^−2^) sensitivity for AA oxidation, and 112 times (619.7 μA mM^−1^ cm^−2^) sensitivity for H_2_O_2_ reduction, respectively, compared to those of pure WO_3_ NFs. These results thus suggest that RuO_2_ NRs-WO_3_ NFs could be a promising candidate electrocatalyst for the fabrication of an efficient electrochemical sensor due to its highly effective electrochemical performance.

## Figures and Tables

**Table 1:** Comparison of the analytical performances of previous reported Ru-based AA sensors.

Electrodes	Methods	Solutions	Potential /V	Sensitivity /μAmM^−1^ cm^2^	Linear Range /μM
RuO_2_ NRs-WO_3_ NFs ^1^	Amperometry	PBS (pH 7.4)	0	171.7	5–2000
RuO_2_-Co_3_O_4_ hybrid nanotubes ^2^	Amperometry	PBS (pH 7.4)	0.05	204	~500
RuO_2_NWs-TiO_2_NFs ^3^	Amperometry	PBS (pH 7.4)	0.018	268.2	10–1500
hAu-Ru nanoshells ^4^	Amperometry	PBS (pH 7.4)	0.05	426	5–2000
AC-RuON-GCE ^5^	DPV	PBS (pH 7.0)	−0.053	85.9	47–181.8
Screen-printing RuO_2_^6^	Amperometry	PBS (pH 7.4)	0.058	2.79	0–4000

^1^ This work, ^2^ Ref. [3], ^3^ Ref. [13], ^4^ Ref. [41], ^5^ Ref. [42], ^6^ Ref, [43].

**Table 2:** Summary of the analytical performances of reported Ru-based H_2_O_2_ sensors.

Electrodes	Methods	Solutions	Potential /V	Sensitivity /μA mM^−1^ cm^−2^	Linear Range /μM
RuO_2_ NRs-WO_3_ NFs ^1^	Amperometry	0.1 M PBS	−0.2	619.7	5–2000
RuO_2_-ReO_3_ (0.11) ^2^	Amperometry	0.1 M PBS	−0.2	667.8	0–5000
RuO_2_NNs-TiO_2_ NRs ^3^	Amperometry	0.05M PBS	0	53.8	1–1000
RuO_2_ NWs-Rh_2_O_3_ NF ^4^	Amperometry	0.05 M PBS	0.12	283.1	0–1000
HRP/Chi-GAD/RuNPs ^5^	Amperometry	Saturated PBS	−0.3	0.798	5090–15,000
Nafion-RuO_2_-AuNP flim ^6^	Amperometry	PBS	−0.4	15.44	0.001–30,000

^1^ This work, ^2^ Ref. [1], ^3^ Ref. [2], ^4^ Ref. [14], ^5^ Ref. [44], ^6^ Ref, [45]. HRP: horseradish peroxidase, Chi: chitosan, GAD: glutaraldehyde

## Supplementary Materials

The followings are available online at https://www.mdpi.com/1424-8220/19/15/3295/s1, Figure S1: EDS elemental mappings, Figure S2: Cyclic voltammograms, Figure S3: Nyquist plots, Figure S4: Amperometric response.
