# Supplementary material for: An Efficient Electrochemical Sensor Driven by Hierarchical Hetero-Nanostructures Consisting of RuO2 Nanorods on WO3 Nanofibers for Detecting Biologically Relevant Molecules
Source: Sensors (Basel). 2019 Jul 26;19(15):3295. doi: 10.3390/s19153295 (PMC6696473; doi:10.3390/s19153295)
Supplement: Supplementary file 1 [file sensors-19-03295-s001.pdf]

Supporting Information

An efficient electrochemical sensor driven by hierarchical hetero-nanostructures consisting of RuO_2 nanorods on WO_3 nanofibers for detecting biologically relevant molecules

Hyerim Lee †, Yeomin Kim †, Areum Yu, Dasol Jin, Ara Jo, Youngmi Lee *, Myung Hwa Kim * and Chongmok Lee *

Department of Chemistry & Nanoscience, Ewha Womans University, Seoul, 03760, Korea

* Correspondence: youngmilee@ewha.ac.kr (Y.L.), myungkim@ewha.ac.kr (M.H.K.), cmlee@ewha.ac.kr (C.L.); Tel.: +82-2-3277-6652 (Y.L.), +82-2-3277-2344 (C.L.), +82-2-3277-4131 (M.H.K.)

† Authors contribute this work equally.

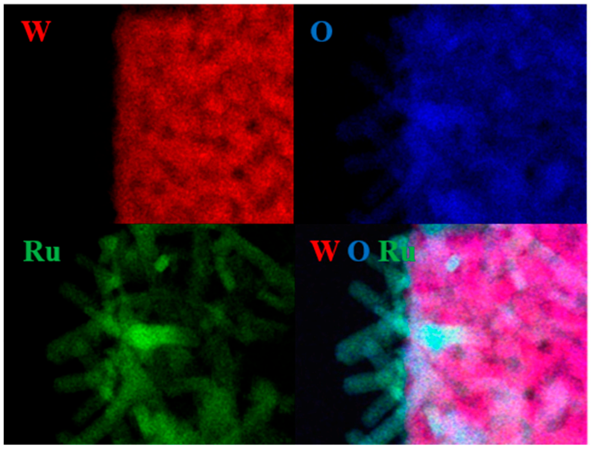

Figure S1. EDS elemental mappings for RuO_2 NRs-WO_3 NFs.

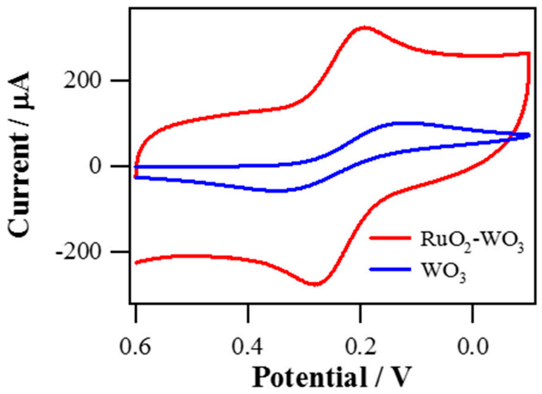

Figure S2. Cyclic voltammograms of RuO_2 NRs-WO_3 NFs and WO_3 NFs in 10 mM $[Fe(CN)_6]^{3-}$ aqueous solution containing 1 M KCl at a scan rate 100 mV s^{-1}.

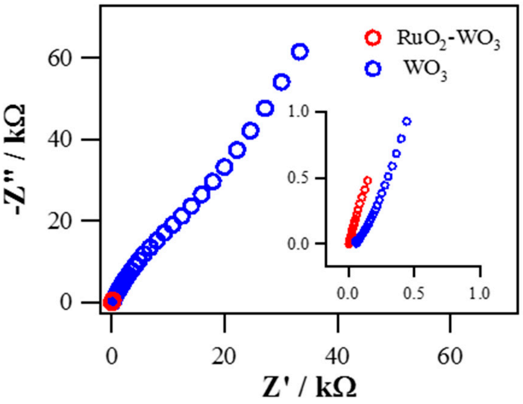

Figure S3. Nyquist plots of RuO_2 NRs-WO_3 NFs and WO_3 NFs in 1 M H_2SO_4 solution with a frequency range of 0.1 Hz – 1000 kHz at 0.5 V (vs. S.C.E.).

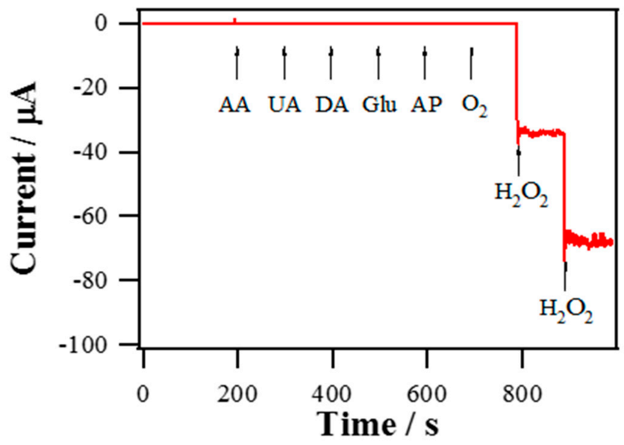

Figure S4. Amperometric response of RuO_2 NRs-WO_3 NFs to sequential additions of 0.1 mM AA, 0.1 mM UA, 0.1 µM DA, 5 mM glucose, 0.1 mM AP, 30 µM O_2, 0.5 mM H_2O_2 and 1 mM H_2O_2 to 0.1 M PBS (pH 7.4) with E_{app} = –0.2 V (vs. S.C.E.).
